# Effect of remineralizing agents on bond strength of orthodontic brackets: an *in vitro* study

**DOI:** 10.1186/s40510-014-0028-y

**Published:** 2014-04-16

**Authors:** Kapil A Ladhe, Murlidhar R Sastri, Jyoti B Madaan, Ketan K Vakil

**Affiliations:** 1 Chalisgaon 424101, MH, India; 2 Department of Orthodontics, S.M.B.T. Dental College and Hospital, Sangamner 422608, MH, India; 3 Diplomate of Indian Board of Orthodontics, Chennai 600040, India

**Keywords:** Casein phosphopeptide amorphous calcium phosphate, Casein phosphopeptide amorphous calcium phosphate with fluoride, Orthodontic brackets, Light-cure adhesive, Chemical-cure adhesive

## Abstract

**Background:**

The purpose of this study is to evaluate the effect of casein phosphopeptide amorphous calcium phosphate (CPP-ACP) and CPP-ACP with fluoride (CPP-ACP-F) on the shear bond strength (SBS) of orthodontic brackets bonded with two different adhesive systems.

**Methods:**

One hundred twenty-six human premolar teeth were selected. One hundred twenty teeth were used for SBS testing, and six teeth were used for scanning electron microscope (SEM) examination. One hundred twenty premolars were divided into mainly three groups: CPP-ACP (group A), CPP-ACP-F (group B), and control group (group C). Each group was sub-divided into two groups according to the bonding adhesive, light cure (groups A1, B1, and C1) and chemical cure (groups A2, B2, and C2). The teeth were pre-treated with the group-specified preventive agent 1 h/day for five consecutive days. Standard edgewise brackets were bonded with the respective adhesives. SBS evaluation was done with the universal testing machine. After debonding, all the teeth were scored for adhesive remaining on the buccal surface, in accordance to adhesive remnant index, under a stereomicroscope. The acid-etched enamel surfaces were observed under SEM after treatment with CPP-ACP, CPP-ACP-F, and artificial saliva.

**Result:**

In light-cure adhesive group, CPP-ACP-F (B1) showed superior results compared to the control group (C1), whereas the CPP-ACP group (A1) showed lower mean SBS than the control group (C1). Both these differences were not statistically significant (*p* > 0.05). In chemical-cure adhesive group, control group C2 showed significantly superior results (*p* < 0.05) compared to group A2 and group B2. The results of two-way ANOVA showed highly significant difference due to adhesive types (*p* < 0.01), whereas enamel pre-treatment showed non-significant difference (*p* > 0.01).

**Conclusion:**

The SBS of the orthodontic brackets was non-significantly affected when the brackets were cured with light-cure bonding system and treated with either CPP-ACP or CPP-ACP-F, whereas with chemical-cure adhesive, decreased bond strength was seen, which was within the clinically acceptable limits.

## 1 Background

Despite the advances in orthodontic materials and techniques in recent years, the development of white spot lesion around the brackets during orthodontic treatment continues to be a problem. Enamel decalcification or white spot lesion is an optical phenomenon which increases in whiteness when dried by air [1]. Nearly 50% of orthodontic patients exhibit clinically visible white spot lesions during treatment that lasts approximately for 2 years, while smooth surface lesions increase up to 50% in prevalence during treatment [2]. Fluoride-containing dentifrice and fluoridated mouthwash has shown to decrease the white spot lesion in orthodontic patients [3]. Also, fluoride may promote the remineralization of white spot lesions if adequate amount of calcium and phosphorus is present in saliva or plaque. For every two fluoride ions, ten calcium ions and six phosphate ions are required to form one unit cell of fluorapatite (Ca_10_(PO_4_)_6_ F_2_) [4]. Hence, on topical application of fluoride ions, the availability of calcium and phosphate ions can be a limiting factor for net enamel remineralization to occur, and this is exacerbated under xerostomic conditions.

A newly developed calcium phosphate remineralization technology based on casein phosphopeptide-stabilized amorphous calcium phosphate (CPP-ACP) stabilizes high concentrations of calcium and phosphate ions, together with fluoride ions, in an amorphous state, at the tooth surface by binding to pellicle and plaque [5]. The proposed mechanism of CPP-ACP anticariogenicity property is that it acts as a calcium phosphate reservoir, buffering the activities of free calcium and phosphate ions in the plaque helping to maintain a state of super saturation with respect to enamel minerals, thereby depressing enamel demineralization and enhancing remineralization [4-8]. Many studies conducted have shown the synergistic effect between CPP-ACP and fluoride which can be attributed to the formation of CPP-stabilized amorphous calcium fluoride phosphate, resulting in the increased incorporation of fluoride ions into plaque, together with increased concentrations of bioavailable calcium and phosphate ions [4,5,9,10].

Very limited studies have been found to be conducted to evaluate the effect of fluoride and CPP-ACP on the bond strength. Also, studies conducted have shown controversial results. In a study conducted by Damon et al. [11] and Bishara et al. [12], the shear bond strength was not affected by various concentrations and methods of application of fluoride. However, the study conducted by Tabrizi and Cakirer [13] concluded that no significant difference was seen between control, CPP-ACP, and CPP-ACP with fluoride group, while fluoride application caused a significant decrease in the tensile bond strength of etch and rinse bonding technique. Kecik et al. [14] compared the effects of CPP-ACP and acidulated phosphate fluoride on SBS values and found higher SBS values for all test groups. Xiaojun et al. [15] reported higher SBS in the CPP-ACP applied group when light-cure adhesives were used. In a study conducted on demineralized enamel by Uysal et al. [16], fluoride and CPP-ACP enhanced the bond strength of the orthodontic brackets compared to the control group in demineralized enamel. In contradiction to this, Ekizer et al. [17] showed no significant difference in fluoride group and control group, while CPP-ACP enhanced the bond strength of the orthodontic brackets.

Therefore, the purpose of this research was to evaluate the effect of CPP-ACP and CPP-ACP with fluoride (CPP-ACP-F) on the shear bond strength (SBS) of orthodontic brackets bonded with two different adhesive systems. The null hypothesis was that pre-treatment with CPP-ACP and CPP-ACP-F would have no effect on the SBS of the orthodontic brackets to enamel with either of the bonding systems.

## 2 Methods

One hundred and twenty-six extracted human premolars (66 maxillary premolars and 60 mandibular premolars) were collected from the Oral and Maxillofacial Surgery Department, S.M.B.T. Dental College and Hospital, Sangamner, India. The teeth extracted were from the patients whose treatment plan needed orthodontic extractions and were collected with the informed consent of the patients. The exclusion criteria for selection of the samples were the teeth with caries, cracks, erosion, fluorosis or hypo-calcification, and restored teeth. One hundred twenty teeth were used for SBS testing, and six teeth were used for SEM examination. One hundred twenty selected teeth were randomly divided into three main groups: group A, group B, and group C (*n* = 40), which were further sub-divided into two groups (*n* = 20) each, depending on the bonding adhesive used, that is, light cure and chemical cure.

The six groups (*n* = 20) with respect to enamel pre-treatments and adhesive systems employed were the following:

*Group A1*: Buccal surface of the crown treated with CPP-ACP (GC Tooth Mousse, GC Corp., Tokyo, Japan) and brackets bonded with light-cure adhesive (Transbond XT, 3 M Unitek, Monrovia, CA, USA).

*Group B1*: Buccal surface of the crown treated with CPP-ACP-F (GC Tooth Mousse Plus, GC Corp.) and brackets bonded with light-cure adhesive.

*Group C1*: No enamel pre-treatment and brackets bonded with light-cure adhesive (control group for light-cure adhesive system)

*Group A2*: Buccal surface of the crown treated with CPP-ACP and brackets bonded with chemical-cure adhesive (Unite Bonding Adhesive, 3 M Unitek).

*Group B2*: Buccal surface of the crown treated with CPP-ACP-F and brackets bonded with chemical-cure adhesive.

*Group C2*: No enamel pre-treatment and brackets bonded with chemical-cure adhesive (control group for chemical-cure adhesive system).

### 2.1 Enamel pre-treatment

The teeth in the CPP-ACP groups (groups A1 and A2) were treated with CPP-ACP diluted tenfold in artificial saliva 1 h/day for 5 days. Similar treatment was done with the CPP-ACP-F groups (groups B1 and B2). The teeth in both control groups (groups C1 and C2) were kept in artificial saliva, and no pre-treatment was done. The components of artificial saliva are given in Table 1.

**Table 1:** Components of the artificial saliva

**Components**	**Weight percent**
NaCl	0.08
KCl	0.12
MgCl_2_ · 6H_2_O	0.01
K_2_HPO_4_	0.03
CaCl_2_ · 2H_2_O	0.01
Sodium carboxy methyl cellulose	0.10
De-ionized water	99.6

### 2.2 Bonding

Before bonding, all teeth were cleaned with non-fluoridated pumice (Glaze polishing paste, Deepti Dental Products, Ratnagiri, Maharashtra, India), treated with 37% phosphoric acid (N-Etch, Ivoclar Vivadent Inc., Amherst, NY, USA) for 30 s, rinsed using an air water syringe for 10 s, and dried until with frosty white appearance.

### 2.3 Bonding with light cure group

The primer was applied only over the desiccated surface, was air-thinned, and light-cured with the help of Hilux 200 halogen light curing machine (Heraeus Kulzer, Benlioglu Dental Inc., Ankara, Turkey) for 10 s. The adhesive paste was applied to the base of the bracket, which was placed on the center of the tooth surface with firm pressure. Excessive adhesive around the bracket was removed. The teeth were again light-cured from all four sides of the bracket, i.e., mesial, distal, occlusal, and gingival, for 10 s each.

### 2.4 Bonding with chemical cure group

A thin layer of primer was applied over the desiccated surface and on the bracket base with a micro-brush. The chemical-cure adhesive was applied to the base of the bracket, which was placed on the center of the tooth surface with firm pressure. Excessive adhesive around the bracket was removed.

### 2.5 Shear bond strength testing

**Figure 1:**
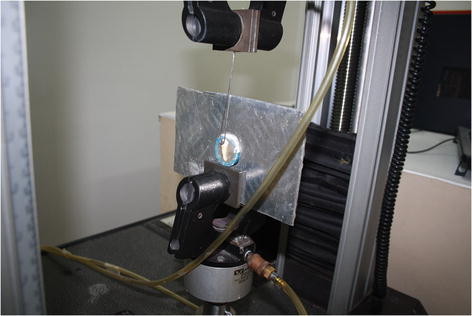
**Specimens were mounted on jig secured to the lower jaw of universal testing machine.** A 0.03-in wire was attached to the upper jaw which applied shear forces at a crosshead speed of 3 mm/min.

For SBS testing, the teeth were embedded in chemical cure acrylic resin so that the buccal surface of each tooth was parallel to the bottom of the mold. A shear force was applied with wire attached to the arm of the universal testing machine (Instron model no. 33R 4467, Instron Ltd., Buckinghamshire, England) at a crosshead speed of 3 mm/min until the brackets were debonded (Figure 1).

### 2.6 Assessment of adhesive remnant index

After debonding, all the samples were examined under a stereomicroscope (Magnus, Olympus India Pvt. Ltd, New Delhi, India) at × 20 magnification to assess the adhesive remnants on the tooth surfaces. The scoring criteria [15] for evaluation are given in Table 2.

**Table 2:** Adhesive remnant index scoring criteria

**Score**	**Criteria**
1	All adhesive, with tooth impression of the bracket base, remained on the teeth.
2	More than 90% of the adhesive remained on the tooth.
3	More than 10% but less than 90% of the adhesive remained on the tooth.
4	Less than 10% of the adhesive remained on the tooth.
5	No adhesive remained on the tooth.

### 2.7 Scanning electron microscope observations

Six premolars were used for ultra-structural examination of the etched enamel surfaces using a scanning electron microscope (SEM). The crowns were sectioned from the roots with a diamond bur at the buccal cementoenamel junction, and each crown was cut longitudinally in an occlusogingival direction to obtain two buccal enamel surfaces. Each surface obtained from the same tooth was randomly allocated to one of three experimental groups: CPP-ACP, CPP-ACP-F, and control group. The teeth were pre-treated as previously explained for each group, etched with the 37% phosphoric acid for 30 s, then rinsed with water for 10 s, and dried until desiccated. The samples were then scanned under the environmental scanning electron microscope (Quanta 200, FEI, Brno, Czech Republic) under low vacuum pressure with × 2,000 magnification.

### 2.8 Statistical analysis

Descriptive statistics including the mean, standard deviation, minimum, and maximum shear bond strength values were calculated for each of the six experimental groups tested. Two-way analysis of variance (ANOVA) was used to determine whether significant differences existed between the bond strength values of the various groups, depending on enamel pre-treatment and adhesive type. A *post hoc* Fisher’s least significant difference (LSD) test was used to determine whether significant differences existed in the bond strength values between the groups. Chi-squared test was used to determine significant differences in the adhesive remnant score. A *p* value less than 0.05 was considered statistically significant.

## 3 Result

Descriptive table of the mean values of SBS with standard deviation, minimum, and maximum values of SBS for all the groups and results of *post hoc* LSD test is presented in Table 3. In light-cure adhesive group, CPP-ACP-F (B1) showed superior results compared to the control group (C1), whereas the CPP-ACP group (A1) showed lower mean SBS than the control group (C1). Both of these differences were not statistically significant (*p* > 0.05). In the chemical-cure adhesive group, control group C2 showed significantly superior results (*p* < 0.05) compared to group A2 and group B2, while group A2 showed better SBS compared to group B2, but was non-significant (*p* > 0.05). Significantly higher SBS values were observed with the light cure group when compared with the chemical cure group. However, the control group (C1 and C2) showed non-significant difference in light cure and chemical cure SBS. Two-way analysis showed that there were significant differences due to adhesive types (*p* < 0.01), whereas non-significant difference was seen due to enamel treatment (*p* > 0.05).

**Table 3:** Descriptive statistics and results of LSD test for the six groups

**Groups**	**Number**	**Shear bond strength**				**Multiple comparison (LSD test),** ***p*** **value**					
		**Mean**	**S.D.**	**Min**	**Max**	**A1**	**B1**	**C1**	**A2**	**B2**	**C2**
		**(MPa)**		**(MPa)**	**(MPa)**						
A1	20	9.76	3.33	4.12	14.77	-	0.030*	0.388	0.035*	0.024*	0.738
B1	20	12.07	2.96	6.22	19.44		-	0.187	0.000*	0.000*	0.065
C1	20	10.67	4.60	5.76	20.23			-	0.003*	0.002*	0.596
A2	20	7.52	1.51	4.46	10.20				-	0.876	0.015*
B2	20	7.36	2.54	3.08	10.38					-	0.010*
C2	20	10.12	4.04	4.91	16.69						-

N, sample size; SD, standard deviation; Min, minimum; Max, maximum; A1, CPP-ACP bonded with light cure; B1, CPP-ACP-F bonded with light cure; C1, control bonded with light cure; A2, CPP-ACP bonded with chemical cure; B2, CPP-ACP-F bonded with chemical cure; C2, control bonded with chemical cure.

*p values <0.05 (significant).

**Figure 2:**
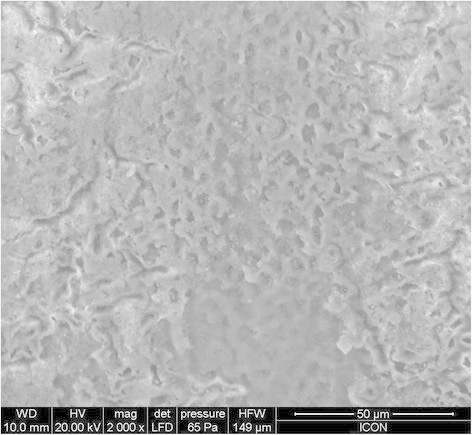
SEM observation of acid-etched enamel after pre-treatment with CPP-ACP.

**Figure 3:**
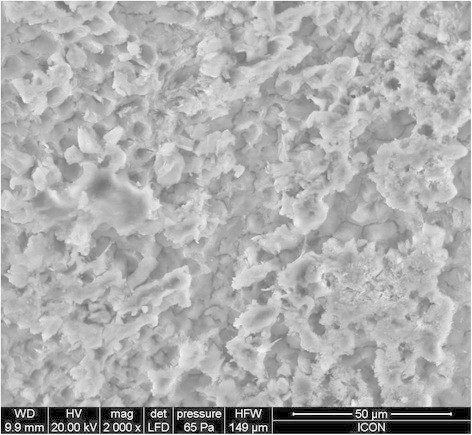
SEM observation of acid-etched enamel after pre-treatment with CPP-ACP with fluoride.

**Figure 4:**
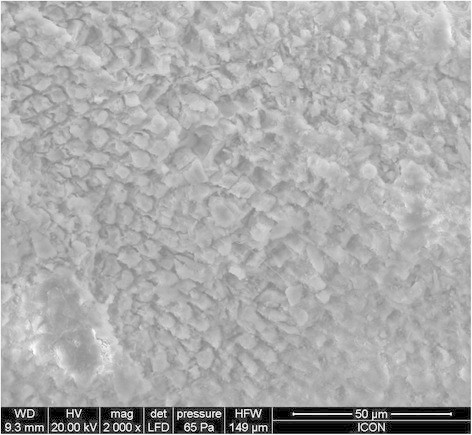
SEM observation of acid-etched enamel without any pre-treatment.

The evaluation of the adhesive remnant index (Table 4) revealed that the bond failure in groups A, B, and C was more in the range of 3 to 4. The SEM images of group A, group B, and group C are given in Figures 2, 3, and 4, respectively. On observation of the etching surface under SEM, group A and group B showed type-III pattern, whereas group C showed type-II enamel etching pattern.

**Table 4:** Frequency table for ARI

**Groups**	**ARI score**				
	**1**	**2**	**3**	**4**	**5**
A1	1 (5%)	0	10 (50%)	8 (40%)	1 (5%)
B1	4 (20%)	2 (10%)	3 (3%)	10 (50%)	1 (5%)
C1	3 (15%)	1 (5%)	8 (40%)	6 (30%)	2 (20%)
A2	0	0	7 (35%)	7 (35%)	6 (30%)
B2	0	0	7 (35%)	9 (45%)	4 (20%)
C2	1 (5%)	2 (10%)	8 (40%)	9 (45%)	0

Value of *χ*^2^ = 47.25, *d.f.* = 20, significant difference (*p* < 0.05).

## 4 Discussion

The plaque retentive properties of the fixed appliances predispose the patient to an increased cariogenic risk [18]. Recently, many authors have advocated CPP-ACP and CPP-ACP-F as the preventive agents for white spot lesions occurring during orthodontic treatment [19,20]. In present literature, many studies have evaluated the effect of CPP-ACP on the SBS of orthodontic brackets, but very limited studies are conducted to evaluate the effect of combination of these two agents on the SBS of orthodontic brackets [13,14,17,21]. So, this study was conducted to evaluate the effect of CPP-ACP and CPP-ACP-F on the SBS of orthodontic brackets bonded with two different adhesive systems.

Light-cure bonding system was included in the study because it is the most commonly used bonding material, whereas chemical-cure adhesive was included because even today, many of the clinicians use chemical-cure bonding system, and there are limited studies [15] evaluating the effects of such remineralizing agents with this system. The group size of 20 teeth each was determined to draw the valid conclusion from *in vitro* bond strength testing [22].

During pre-treatment, CPP-ACP and CPP-ACP-F were diluted tenfold in artificial saliva in order to simulate the oral environment. For dilution, the artificial saliva was selected because the product manual for Tooth Mousse [23] emphasized that saliva would enhance the effectiveness of CPP-ACP, and the longer CPP-ACP and saliva are maintained in the mouth, the more effective the result is.

A universal testing machine was used to apply a shear-type stress on the specimen at the crosshead speed set to 3 mm/min which is preferred commonly in laboratory studies. The shear bond testing was with the help of a loop wire because Mojtahedzadeh et al. [24] in their study mentioned that the loop wire method has more similarity to clinical loads and lower dispersion of values than the blade method, for debonding of brackets in a shear mode.

In the present study, the treatment with CPP-ACP and the use of light-cure adhesive showed slightly inferior results as compared to the control group, but this difference was non-significant. This finding was slightly different from the previous study done by Tabrizi and Cakirer [13] who showed non-significant increase in mean SBS. However, this findings were in clear contradiction to the other studies done by Kecik et al. [14] and Xiaojun et al. [15] who showed that topical application of CPP-ACP enhanced the bond strength significantly. The effect of the CPP-ACP on teeth bonded with chemical-cure adhesive system showed significant decrease in the mean SBS compared to that of the control group. This was in contradiction to the previous study of Xiaojun et al. [15] who reported non-significant increase in the SBS with CPP-ACP pre-treatment and bonded with chemical-cure adhesive systems.

The effect of CPP-ACP-F bonded with light-cure adhesive system in the present study showed non-significant increase in the SBS compared to that of the control group. This finding was in agreement to the previous study of Tabrizi and Cakirer [13] who reported no significant difference between the comparative groups, while this finding was in contradiction to another study done by Ekizer et al. [17] who evaluated the effect of different demineralization inhibition methods on the SBS of orthodontic brackets. In the group treated with CPP-ACP-F and bonded with chemical-cure adhesive system, there was a significant decrease in the bond strength compared to the control group. No previous study was found in the literature evaluating the effect of CPP-ACP-F on the SBS of orthodontic bracket bonded with chemical-cure adhesive.

The shear bond strength of orthodontic brackets bonded with chemical and light-cure adhesives was in the range from 7 to 12 MPa. This range is higher than the recommended range of 6 to 8 MPa, which is adequate for orthodontic purpose [25].

The ARI score in all six groups was in the range of 3 to 4, indicating a minimum amount of adhesive that remained on the debonded tooth surface. When the etched surface was observed under SEM, group A and group B showed more roughened surface compared to group C. This increased bond surface area may be the reason for the increased bond strength of the light cure group.

## 5 Conclusion

Careful interpretation of the findings led to the following conclusions:

1. CPP-ACP and CPP-ACP with fluoride showed no significant effect on the shear bond strength of the orthodontic brackets when bonded with light-cure adhesive. This indicated the safety of these products in caries prophylaxis in orthodontic patients without compromising shear bond strength of the orthodontic brackets.

2. In chemical-cure adhesive, the pre-treatment with CPP-ACP and CPP-ACP with fluoride showed significantly decreased shear bond strength of the orthodontic brackets. However, the mean shear bond strength was clinically acceptable.

3. Significant difference was seen in the ARI scores between all the groups.

## Competing interests

The authors declare that they have no competing interests.

## Authors’ contributions

KAL conducted the *in vitro* study and drafted the manuscript. MRS revised the draft critically for important intellectual content. JBM helped draft the manuscript and revised it thoroughly. KKV supervised all the procedures and helped in finalizing the manuscript. All authors read and approved the final manuscript.
